# The 2021 AHA/ACC Guideline for the Evaluation and Diagnosis of Chest Pain: An Interventionalist’s Viewpoint

**DOI:** 10.1016/j.jscai.2022.100305

**Published:** 2022-04-08

**Authors:** Robert F. Riley, Wayne B. Batchelor, James A. Goldstein, Rasha Al-Lamee, Samit Shah, Jennifer A. Tremmel, Farouc Jaffer, Timothy D. Henry

**Affiliations:** a Overlake Medical Center & Clinics, Bellevue, Washington; b Inova Heart and Vascular Institute, Fairfax, Virginia; c Beaumont Hospital, Royal Oak, Michigan; d Imperial College Healthcare NHS Trust, London, United Kingdom; e Yale School of Medicine, New Haven, Connecticut; f Stanford University Medical Center, Stanford, California; g Massachusetts General Hospital/Harvard Medical School, Boston, Massachusetts; h The Carl and Edyth Lindner Center for Research and Education at The Christ Hospital, Cincinnati, Ohio

**Keywords:** Stress testing, chest pain, coronary artery disease

Chest pain is one of the most common chief complaints in both inpatient and outpatient settings in the United States. Given that coronary artery disease (CAD) remains the leading cause of death in the United States, discriminating between patients presenting with actionable cardiac disease vs those with other “noncardiac” etiologies for their symptoms is of the utmost importance. Over the past 5-10 ​years, we have seen a rise in various biomarkers (eg, high-sensitivity troponins), clinical decision pathways (eg, HEART score), and novel diagnostic imaging modalities (eg, coronary computed tomography angiography [CCTA]) to improve diagnostic discrimination; however, there is considerable care variability in clinical practice across the United States. To help standardize this process, the American College of Cardiology and American Heart Association Joint Committee on Clinical Practice Guidelines released a guideline document on the evaluation and diagnosis of chest pain.^1^ Many of the central themes of this document align with prior guideline recommendations, although there are some nuanced central themes worth noting (Figure 1). Based on a recent SCAI webinar on this topic, we have highlighted the key areas of the guideline most likely to impact interventional cardiologists.

**Figure 1:**
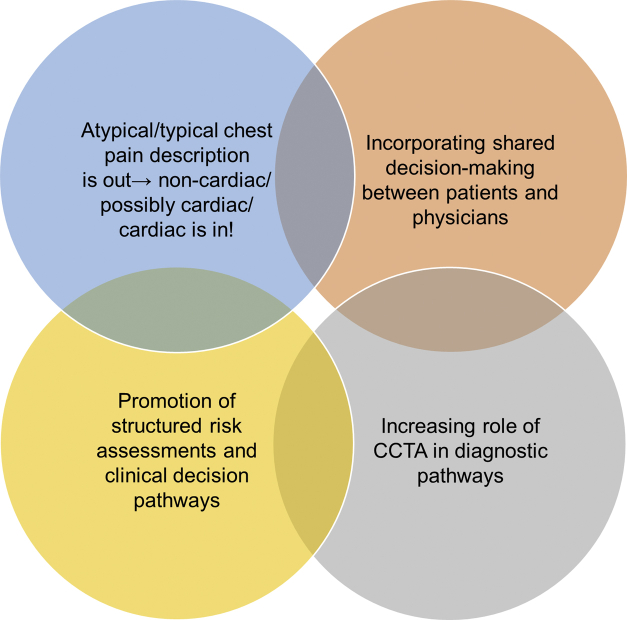
Central themes for the 2021 American Heart Association/American College of Cardiology guideline for the evaluation and diagnosis of chest pain. CCTA, coronary computed tomography angiography.

## Incorporating sex, age, ethnicity, and cultural norms into the evaluation of chest pain

The evaluation of chest pain is often subjective and relies on patient history and physician assessment of symptoms. Within this clinical evaluation, conscious and unconscious biases with regard to sex, age, ethnicity, and/or cultural background can influence the classification of symptoms within the ischemic continuum. In managing the diverse group of patients presenting with chest pain, the guideline provides a framework for the provision of equitable care to improve clinical outcomes.

For example, although women are more likely to present to the emergency department with chest pain, they are less likely to have timely and clinically appropriate care than men.^2^ Women presenting with chest pain are also more frequently labeled as having “noncardiac” pain that is often associated with symptoms such as palpitations and neck pain.^3^ Therefore, for women presenting with chest pain, the guideline recommends obtaining a history that emphasizes accompanying symptoms that are more common in women with acute coronary syndromes (ACS).

The management of elderly patients with chest pain can also be complex because while they have greater risk factors for cardiovascular disease, they also have comorbidities that may lead to misdiagnosis of chest pain as noncardiac.^4^ The guideline recommends that for patients over the age of 75 ​years, atypical presenting features such as a fall, syncope, or acute delirium should be considered to be possible manifestations of ACS.

There are significant disparities in the management of chest pain across ethnic groups as well, with Black and Hispanic patients being far more likely to be undertreated and receive lower quality care than their Caucasian counterparts.^5^ The guideline recommends the introduction of cultural competency training to break down cultural barriers and the use of formal translation processes while managing patients in order to improve the quality of care.

## Recommendations on evaluating acute chest pain

The new guideline places emphasis on the early recognition of acute ischemic symptoms, including nonclassic anginal symptoms, and prompt activation of emergency medical services by the patient dialing 9-1-1. In the emergency department, initial evaluation should focus on rapid identification of patients at highest risk of a cardiac emergency, including those with ACS, aortic dissection, and pulmonary embolism or nonvascular emergencies such as esophageal rupture or pneumothorax. The 12-lead electrocardiogram and preferred high-sensitivity troponin facilitate rapid and accurate detection of myocardial injury, thereby aiding risk assessment. Once acute chest pain is confirmed, stratification into low-, intermediate-, and high-risk categories through the routine use of structured risk assessments can help guide further diagnostic testing, including coronary angiography. Coronary angiography is still recommended for patients with true ACS, hemodynamic instability, and/or a high-risk clinical assessment. Intermediate-risk patients can often undergo functional stress testing or CCTA. Low-risk patients typically require no further testing and can be discharged with close clinical follow-up.^1^

## Recommendations on evaluating chronic/stable chest pain

Evaluating patients with stable, chronic chest pain also relies on risk, although this is less well defined and typically overestimates disease burden compared with those with acute chest pain. As defined in the guideline, the pretest probability calculation is based on age, sex, and symptom characterization and can be augmented by a coronary artery calcium score if available. For low-risk patients with stable symptoms, a coronary artery calcium score can be useful in selected cases to exclude the presence of calcified CAD, although an exercise treadmill test can also be useful in this group. For those patients determined to be at intermediate-to-high risk for CAD, CCTA or stress imaging is recommended, including fractional flow reserve computed tomography (FFR-CT) when available for those found to have intermediate stenoses during CCTA. Of note, in this guideline, if nuclear stress imaging is selected, positron emission tomography is preferred over single-photon emission computerized tomography to improve diagnostic accuracy and decrease the rate of nondiagnostic results (2a, B-R recommendation).

In those with intermediate-to-high pretest probabilities for disease, it is always important to remember that CCTA or coronary angiography can be utilized in those patients with negative or inconclusive stress imaging studies and/or ongoing symptoms without explanation. Additionally, coronary angiography is indicated with those patients with documented ischemia and ongoing symptoms despite optimal medical therapy (OMT), FFR-CT ≤0.8, or high-risk anatomy seen on CCTA.

## Ischemia with nonobstructive coronary arteries

For patients with ongoing symptoms and no clear explanation, evaluation for ischemia with nonobstructive coronary arteries (INOCA) may be appropriate. In applying these recommendations to INOCA patients, we must remember that (1) approximately half have “nontypical” angina, which does not mean “noncardiac,” nor does it mean not cardiac in etiology, (2) they have recurrent, mostly stable symptoms, so while they may present to the emergency department with a flare, their course is generally chronic rather than acute, (3) pretest probability is used for determining the likelihood of obstructive CAD and is therefore not a relevant guidepost for pursuing further evaluation in these patients, and (4) conventional stress testing is insufficient for identifying who will have an occult coronary cause of their chest pain, so a negative stress test does not preclude further evaluation.^6^, ^7^, ^8^

Thus, regardless of whether a patient’s stress test was positive or negative, for those with ongoing chest pain with exertion, emotional stress, or even when unprovoked, who have nonobstructive coronary arteries by CCTA or coronary angiography, further testing for an occult coronary etiology should be considered. While noninvasive tests, specifically positron emission tomography and cardiovascular magnetic resonance imaging, may assess for microvascular dysfunction, definitive testing falls to the interventional cardiologist, who can perform comprehensive invasive testing for endothelial dysfunction, epicardial spasm, microvascular dysfunction, and/or myocardial bridging, providing a diagnosis that directs management, reduces angina, and improves quality of life.^9^, ^10^, ^11^

## Overview of noninvasive diagnostic testing and implications for interventional cardiologists

As noted above, the guideline recommendations for noninvasive diagnostic testing and coronary angiography utilization are stratified by the pretest probability of CAD and likelihood of detecting ischemia. How should interventional cardiologists interpret these recommendations? Probability-based diagnostic testing of patients presenting with chest pain should improve the diagnostic yield of invasive coronary angiography and can help guide decisions regarding revascularization, though we should be cautious about the possibility of overdiagnosis with anatomic testing of lower-risk patients and underdiagnosis in patients with INOCA.^12^ Regardless, all patients with CAD should receive goal-directed OMT, with revascularization reserved for patients presenting with ACS, highly symptomatic patients despite OMT, and those with high-risk anatomic disease burden.^13^^,^^14^

In conclusion, the guideline provides new evidence-based recommendations for the assessment and evaluation of acute and stable chest pain syndromes. A careful, structured evaluation allows for patients to be categorized into risk profiles. That risk profile then helps determine the diagnostic workup. Invasive coronary angiography remains the gold standard for diagnosing CAD but also enables physiological assessment of the epicardial arteries (FFR and nonhyperemic pressure indices) and microvasculature (coronary flow reserve measurement, microvascular spasm), detection of coronary abnormalities (myocardial bridge, coronary aneurysms, and fistula), and intravascular imaging to diagnose spontaneous coronary artery dissection and plaque rupture/erosion for appropriate patients. Additionally, we will likely continue to see evolution of synergistic noninvasive modalities for identifying many of these coronary diagnoses in the years to come.

## Declaration of competing interest

Dr Robert F. Riley provides consultation for Boston Scientific, Abbott Vascular, Medtronic, and Shockwave Medical. Dr Farouc Jaffer is a speaker in Boston Scientific and provides consultation for Boston Scientific and Siemens Healthineers. Dr Jennifer Tremmel provides consultation for Boston Scientific and Abbott Vascular, is an advisory board member in Boston Scientific and Abbott Vascular, and reports research support from 10.13039/100008497Boston Scientific. The other authors have no conflicts to report.
